# Fate of hips complicated by avascular necrosis of the femoral head following reconstructive surgery in nonambulatory patients with cerebral palsy

**DOI:** 10.1038/s41598-022-16023-7

**Published:** 2022-07-11

**Authors:** Byoung Kyu Park, Hoon Park, Kun Bo Park, Isaac Rhee, Sungmin Kim, Hyun Woo Kim

**Affiliations:** 1 Department of Orthopaedic Surgery, Inje University Haeundae Paik Hospital, Busan, Republic of Korea; 2 Department of Orthopaedic Surgery, Gangnam Severance Hospital, Yonsei University College of Medicine, Seoul, Republic of Korea; 3 Division of Pediatric Orthopaedic Surgery, Severance Children’s Hospital, Yonsei University College of Medicine, 50-1 Yonsei-ro, Seodaemun-gu, Seoul, 03722 Republic of Korea; 4 Department of Orthopaedics, St Vincent’s Hospital, Melbourne, Australia; 5 Department of Orthopaedic Surgery, Chonnam National University Hospital, Gwangju, Republic of Korea

**Keywords:** Neuromuscular disease, Outcomes research, Paediatric research, Paediatric neurological disorders, Encephalopathy, Neuromuscular disease, Neurological disorders, Risk factors

## Abstract

The purpose of this study was to evaluate the influence of avascular necrosis of the femoral head (AVN) following hip reconstructions on the future hip development of cerebral palsy (CP) patients. A retrospective study of 394 hips in 205 nonambulatory patients with spastic CP who underwent reconstructive hip surgery was performed. The mean age at surgery was 7.3 ± 2.4 years. The mean follow-up duration was 5.6 ± 2.7 years, and the mean age at the latest follow-up was 12.8 ± 3.4 years. AVN was classified in terms of its severity and location. Femoral head remodelling was assessed by the spherical index and the Mose circle. An unsatisfactory radiological outcome was defined as having a migration percentage of more than 30% at the final follow-up. AVN was observed in 169 (42.9%) hips. Older age at the time of surgery, higher preoperative migration percentage, and open reduction procedures were predictors for the development of AVN. Hips with AVN confined to the lateral epiphysis, and AVN involving the entire epiphysis with preserved height experienced successful remodelling. 27 (65.9%) of the 41 hips with unsatisfactory outcomes experienced AVN. Younger age, higher postoperative migration percentage, and occurrence of AVN were related to unsatisfactory outcomes. The highest incidence of failed remodelling and unsatisfactory outcomes were observed in hips with entire epiphyseal involvement and more than 50% loss of its height. AVN following hip reconstructions is not necessarily associated with poor hip development, however, depending on the severity and location, it is a prognostic factor for unsatisfactory radiological outcomes.

## Introduction

Avascular necrosis of the femoral head (AVN) is one of the most serious complications following treatment of developmental dysplasia of the hip (DDH) ^1–3^. Any disturbance of the proximal femoral growth may jeopardize long-term outcomes, even in hips with normal acetabular development. Hence, greater caution is used for infants and young children.

On the contrary, less attention is paid to the development of AVN following reconstructive hip surgery in cerebral palsy (CP). The majority of studies simply comment on the presence or absence of AVN in their complications ^4^. Although several studies have reported the potential risk factors of AVN ^5–9^, the relationship between AVN and subsequent development of the hip joint has not been explored in the literature. This is despite the fact that CP has a differing pathophysiology and considerably more severe deformities of the proximal femur and acetabulum than DDH ^10^. Progressive hip displacements are caused by persistent spasticity and soft-tissue contractures around the hip joint. Operative intervention is usually performed in older children, when compared to DDH, at which time there is presence of a more ossified femoral head.

In the present study, we observed the radiographic features of AVN following hip reconstructions in nonambulatory patients with spastic CP and aimed to examine their influences on the patients’ future hip development. Specifically, our objectives were to: (1) examine the severity and location of AVN; (2) identify any clinical or radiographic predictive factors for the development of AVN; and (3) evaluate the developmental hip status in relation to AVN and its subsequent remodelling.

## Methods

### Participants

This retrospective study was approved by the Institutional Review Board of Severance Hospital (IRB No. 4-2018-1185). All methods were carried out in accordance with relevant guidelines and regulations. The Institutional Review Board of our hospital waived the need for informed consent from the patients as our research involved no more than a minimal risk to our subjects and we utilized existing medical records and radiographs. We reviewed the medical records and pelvic radiographs of 259 consecutive patients with spastic CP who underwent one-stage reconstructive hip surgery between 2001 and 2015. The surgical indications included patients with impaired sitting balance and perineal care due to their long-standing hip adductor and flexor contractures.

The exclusion criteria included: (1) 4 patients who underwent an initial surgery elsewhere and were later referred for recurrent hip subluxation or dislocation; (2) 18 ambulatory patients with Gross Motor Function Classification System (GMFCS) ^11^ level II or III, who underwent different hip reconstruction procedures from the nonambulatory; and (3) 32 patients whose preoperative and/or follow-up radiographs were not available in our current electronic medical imaging system.

A total of 394 hips in 205 patients with GMFCS level IV or V were included in the study. Patient demographics are summarized in Table 1. There were 120 males and 85 females, and the mean age at the time of surgery was 7.3 ± 2.4 years. The mean duration of follow-up was 5.6 ± 2.7 years, and the mean age at the final follow-up was 12.8 ± 3.4 years. One hundred and eighty-nine patients (92.2%) had bilateral surgeries, and 16 unilateral. Varus derotational osteotomy of the proximal femur (VDRO) was performed in all cases. Open reduction of the femoral head was performed in 128 hips (32.5%), and a pelvic osteotomy was completed in 210 hips (53.3%). Ninety-nine hips (25.1%) underwent simultaneous open reduction and pelvic osteotomy. To prevent further hip displacement and improve patient’s hip symmetricity and sitting balance, hip reconstructions were performed in 49 spastic contralateral hips with a migration percentage of less than 30%.

**Table 1:** Data on the patients.

Parameters	Values
Number of patients	205
**Sex**	
Male (%)	120 (58.5)
Female (%)	85 (41.5)
Bilateral surgery (%)	189 (92.2)
Age at surgery (years, mean ± SD)	7.3 ± 2.4
Duration of follow-up (years, mean ± SD)	5.6 ± 2.7
Age at final follow-up (years, mean ± SD)	12.8 ± 3.4
Number of hips	394
**Side**	
Right (%)	198 (50.3)
Left (%)	196 (49.7)
Open reduction (%)	128 (32.5)
Pelvic osteotomy (%)	210 (53.3)
Open reduction and pelvic osteotomy (%)	99 (25.1)

All operative procedures were performed by the senior author, and the surgical techniques used were similar to those previously described ^12–16^. Tenotomies of the adductor longus and gracilis and neurectomy of the anterior branch of obturator nerve were completed through a small transversal incision made in the groin. Adductor brevis and the medial hamstrings were also released as necessary. The primary surgical treatment for displaced hips was a VDRO at the intertrochanteric level, and the degrees of varus angulation and external rotation of the distal fragment were adjusted to achieve a concentric reduction of the femoral head within the acetabulum. The psoas tendon was completely released and the lesser trochanteric apophysis was excised en bloc. An angled blade plate (DePuy Synthes, West Chester, PA) or a pediatric hip plate (DePuy Synthes, West Chester, PA) was utilized to fix the osteotomy. For severely subluxated or dislocated hips, a traditional open reduction through an anterolateral approach to the hip was performed, before or after VDRO. Dega-type pericapsular acetabuloplasty for the treatment of acetabular dysplasia was carried out to improve acetabular coverage. An incomplete iliac osteotomy was completed just above the open or closed triradiate cartilage and the site was opened using a laminar spreader until the acetabular coverage was sufficiently improved. An iliac allograft bone (Tutoplast^®^ Iliac Crest Wedge, RTI Surgical, Alachua, FL) was trimmed into a triangular or trapezoidal shape after the appropriate length and size was determined, and then inserted and wedged securely into the osteotomy site. The patients were immobilized in a fiberglass hip spica cast after surgery for a total of 4–6 weeks, with the hip in approximately 25° abduction and neutral or slight internal rotation. After removal of the cast, a hip abduction brace was worn during napping hours and overnight for the next 6 months.

### Radiographic analysis and identification of AVN

**Figure 1:**
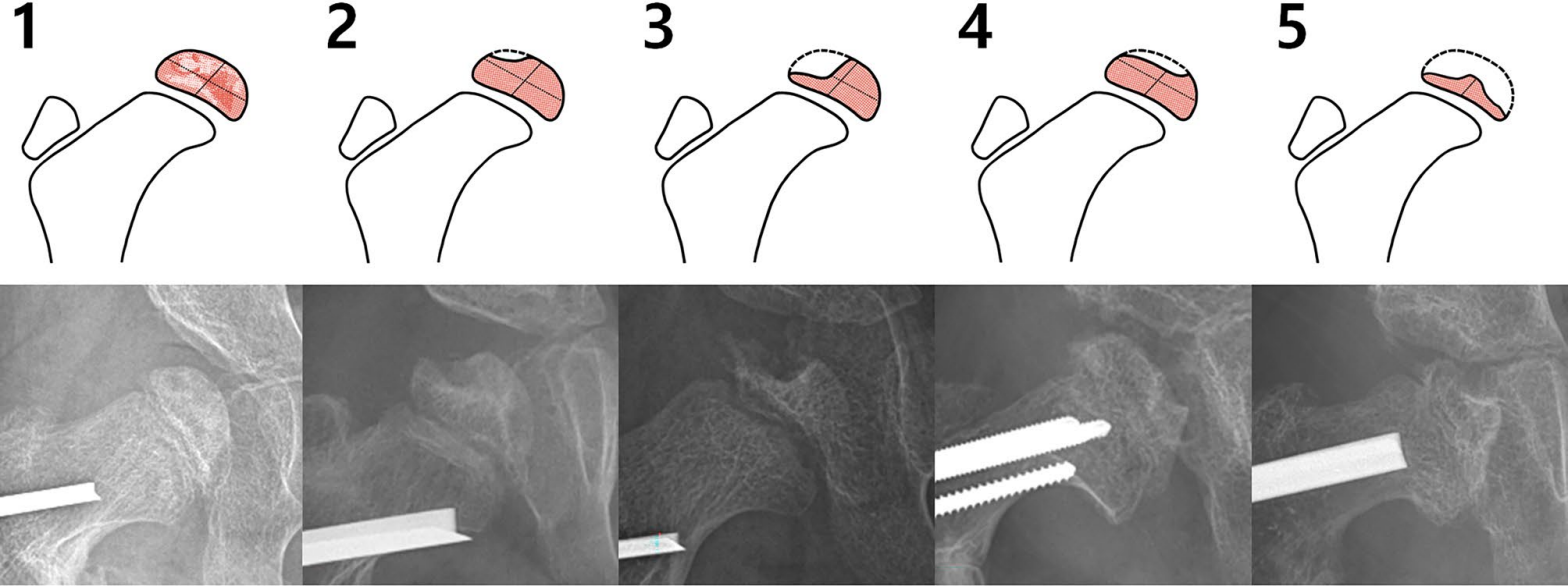
Five-type classification of avascular necrosis of the femoral head (AVN) based on the severity and location. Severity was defined as mild if height was preserved, moderate if more than 50% of height was retained, and severe if less than 50% of epiphyseal height remained. Types 2 and 3 AVN were confined to the lateral epiphysis, and Types 1, 4, and 5 involved the entire epiphysis.

Routine radiological follow-up using standardized anteroposterior pelvic radiographs ^17^ included imaging at the time of cast removal, every 3 months until postoperative year 1, every 6 months until postoperative year two, and then on an annual basis. All serial radiographs were examined for any postoperative radiographic evidence of AVN in the epiphysis. These included localized or diffuse changes in the radio-density, irregular fragmentation, flattening, collapse, and defects. Changes to the epiphysis were classified into five types according to their severity and location (Fig. 1). Severity was categorized into mild (changes in the radio-density but no loss of epiphyseal height), moderate (collapsed epiphysis with preservation of more than 50% of the original height), or severe (preservation of less than 50% of the original epiphyseal height) based on the area that was most severely affected. The location was categorized into either involvement of the lateral one half or the entire epiphysis.

Radiographic parameters for hip integrity ^18–20^ such as the migration percentage, acetabular angle of Sharp, and femoral head-shaft angle were measured at three time-points: before surgery, immediately after surgery, and at the latest follow-up. We examined whether the femoral head with AVN had remodelled at the final follow-up using the spherical index and the Mose method, which were measured immediately after surgery and at the final follow-up. The spherical index ^21^ was used to calculate any flattening of the epiphysis, whilst the Mose criteria ^22^ was applied to evaluate the overall sphericity. The spherical index is calculated using the maximum height and width of the head and is used to evaluate the roundness of the femoral head, however, as it may not reflect the focal resorption of the epiphysis, we also used the Mose method. Failed remodelling of the femoral head after AVN was defined as when both a decrease in the spherical index and variation of more than 2 mm in the Mose criteria were identified. Unsatisfactory radiological outcome was defined as having a migration percentage of more than 30% at the final follow-up ^23^.

### Statistical analysis

All statistical analyses were carried out using SPSS version 25.0 (IBM Corp., Armonk, NY), and a p-value < 0.05 was considered statistically significant. The intra- and inter-rater reliabilities of each radiologic parameter were examined; one of the authors measured the parameters twice at occasions two weeks apart, whist another author measured the same parameters, blinded and independent of the other author’s measurements. Intraclass correlation coefficients were calculated using a two-way random-effect model assuming a single measurement and absolute agreement for continuous variables, and Cohen’s kappa coefficients were calculated for categorical variables.

One-way analysis of variance was used to compare preoperative spherical index among the six groups, and repeated measure analysis of variance was utilized to examine the changes in the migration percentage and head-shaft angle over time. Paired t-tests were used to compare preoperative and final acetabular angle of Sharp and the spherical index. Shapiro–Wilk’s test was applied to check the data distribution. Considering the inclusion of bilateral cases, univariable and multivariable generalized estimating equations (GEE) were used to examine the association of clinical and radiological parameters on the development of AVN as well as for the unsatisfactory outcomes. All potential risk factors were analyzed in each univariable analysis, and the variables identified as significant were included in the multivariable analysis. Odds ratios (OR) with 95% confidence intervals (CI) were calculated. The Fisher exact test was used to compare unsatisfactory outcomes according to the existence of failed remodelling.

## Results

Both the continuous and categorical variables showed good to excellent intra- and inter-rater reliabilities (Table 2) ^24^. All radiographic parameters are summarized in Table 3. The mean migration percentage improved from 67.1 ± 26.0% preoperatively to 11.0 ± 12.7% postoperatively, and it was noted to be increased to 24.2 ± 18.7% at the final follow-up. The head-shaft angle was also corrected after surgery and measured to be increased at the final follow-up. Of the initial 394 hips, 41 (10.4%) were identified as having unsatisfactory outcomes with a migration percentage of more than 30% at the final follow-up. Significant predictors of unsatisfactory outcomes were a younger age at the time of surgery (OR 0.84, 95% CI 0.73 to 0.96, p = 0.013), higher postoperative migration percentage (OR 1.04, 95% CI 1.02 to 1.06, p = 0.001), and occurrence of AVN (OR 2.37, 95% CI 1.03 to 5.44, p = 0.042) (Table 4).

**Table 2:** Intra-rater and inter-rater reliabilities of the radiographic indices.

Indices	Intra-rater reliability		Inter-rater reliability	
	ICC	95% CI	ICC	95% CI
Migration percentage	0.942	0.898 to 0.967	0.946	0.905 to 0.969
Acetabular angle	0.827	0.692 to 0.903	0.776	0.605 to 0.873
Head-shaft angle	0.795	0.640 to 0.883	0.782	0.609 to 0.878
Spherical index	0.833	0.680 to 0.917	0.734	0.510 to 0.865

	Kappa	95% CI	Kappa	95% CI
Type of AVN	0.876	0.762 to 0.990	0.776	0.633 to 0.919

*ICC* intraclass correlation coefficient, *CI* confidence interval, *AVN* avascular necrosis of the femoral head.

**Table 3:** Changes in the radiographic parameters.

Parameters	Preoperative	Postoperative	Final follow-up	p value
Migration percentage (mean ± SD)	67.1 ± 26.0	11.0 ± 12.7	24.2 ± 18.7	< 0.001
Acetabular angle (mean ± SD)	54.2 ± 5.0	N/A	47.2 ± 6.2	< 0.001
Head-shaft angle (mean ± SD)	163.9 ± 11.7	128.4 ± 9.6	137.4 ± 11.3	< 0.001
Spherical index (mean ± SD)	38.0 ± 4.7	N/A	43.7 ± 5.6	< 0.001

**Table 4:** Parameters associated with unsatisfactory outcomes at the final follow-up.

Parameters	Univariable		Multivariable	
	OR (95% CI)	p value	OR (95% CI)	p value
Age at surgery	0.85 (0.73 to 0.99)	0.039	0.84 (0.73 to 0.96)	0.013
Sex	1.14 (0.58 to 2.23)	0.711		
Preoperative migration percentage	1.02 (1.01 to 1.04)	0.001	1.02 (1.00 to 1.04)	0.093
Preoperative acetabular angle	0.98 (0.91 to 1.05)	0.545		
Preoperative head-shaft angle	1.02 (0.98 to 1.06)	0.326		
Open reduction	2.41 (1.22 to 4.78)	0.011	1.55 (0.71 to 3.37)	0.268
Pelvic osteotomy	0.82 (0.42 to 1.61)	0.558		
Postoperative migration percentage	1.03 (1.01 to 1.06)	0.013	1.04 (1.02 to 1.06)	0.001
Postoperative head-shaft angle	1.02 (0.98 to 1.06)	0.338		
Avascular necrosis	2.87 (1.45 to 5.66)	0.002	2.37 (1.03 to 5.44)	0.042

Generalized estimating equations were used to calculate the ORs and CIs; *OR* odds ratio, *CI* confidence interval.

AVN were observed in 169 (42.9%) hips; 64 (37.9%) hips with mild changes, 72 (42.6%) moderate, and 33 (19.5%) severe. The two most common types were Types 1 (64 hips, 37.9%) and 4 (63 hips, 37.3%). Evidence of AVN was seen on an average of 7.5 ± 3.8 months after surgery, and 152 hips (89.9%) showed AVN within the first postoperative year. Among the 17 hips in which AVN were observed after postoperative year 1, 13 had mild changes and 4 moderate. We attempted to investigate the optimal time to identify radiographic changes for determination of each subtype of AVN, however, there was great variation and a specific time could not be determined. Multivariable GEE revealed that an older age at the time of surgery (OR 1.13, 95% CI 1.00 to 1.26, p = 0.042), higher preoperative migration percentage (OR 1.04, 95% CI 1.03 to 1.06, p < 0.001), and open reduction procedures (OR 4.70, 95% CI 2.50 to 8.84, p < 0.001) were significant predictors of AVN (Table 5).

**Table 5:** Parameters associated with development of avascular necrosis of the femoral head.

Parameters	Univariable		Multivariable	
	OR (95% CI)	p value	OR (95% CI)	p value
Age at surgery	1.11 (1.02 to 1.22)	0.019	1.13 (1.00 to 1.26)	0.042
Sex	1.07 (0.67 to 1.71)	0.788		
Preoperative migration percentage	1.05 (1.04 to 1.07)	< 0.001	1.04 (1.03 to 1.06)	< 0.001
Preoperative acetabular angle	1.04 (0.99 to 1.09)	0.102		
Preoperative head-shaft angle	1.01 (0.99 to 1.04)	0.405		
Open reduction	11.65 (6.58 to 20.62)	< 0.001	4.70 (2.50 to 8.84)	< 0.001
Pelvic osteotomy	4.45 (2.76 to 7.19)	< 0.001	1.03 (0.48 to 2.22)	0.939
Postoperative migration percentage	0.97 (0.95 to 0.99)	0.001	0.98 (0.96 to 1.01)	0.120
Postoperative head-shaft angle	1.00 (0.98 to 1.03)	0.729		

Generalized estimating equations were used to calculate the ORs and CIs; *OR* odds ratio, *CI* confidence interval.

**Figure 2:**
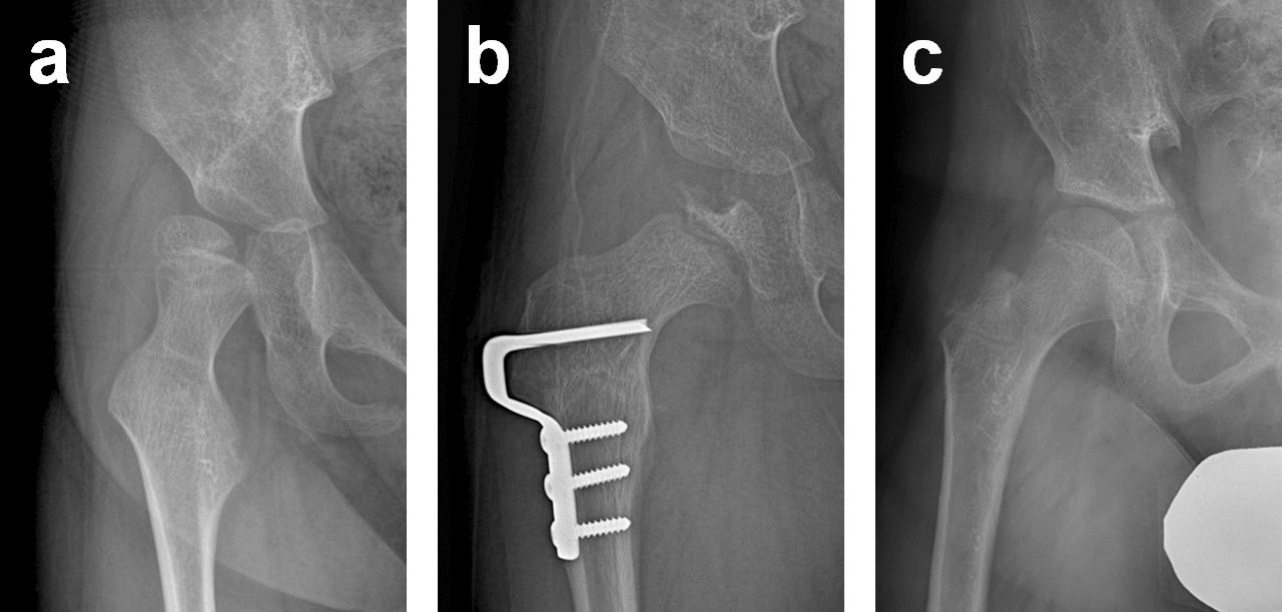
(**a**) Preoperative anteroposterior (AP) radiograph showing a subluxated hip in a 6-year-old boy. (**b**) AP radiograph showing Type 3 AVN developed after open reduction and varus derotational osteotomy of the proximal femur (VDRO). (**c**) At 6 years after surgery, the femoral head was noted to be remodelled without subluxation.

**Figure 3:**
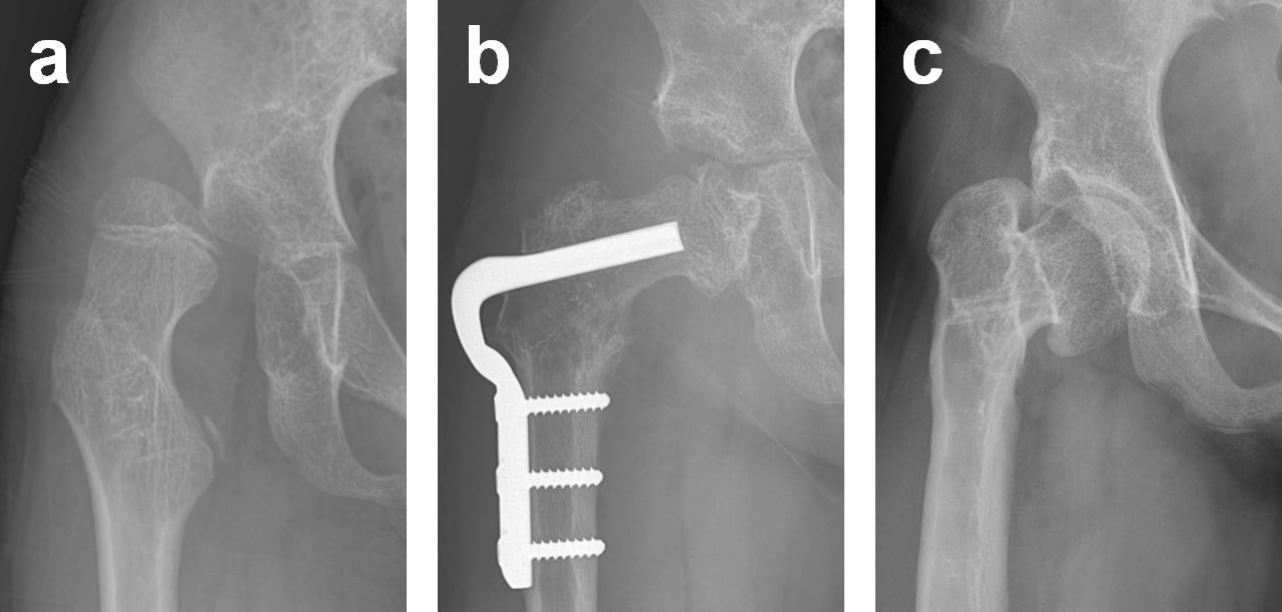
(**a**) Preoperative AP radiograph showing a dislocated hip in an 8-year-old girl. (**b**) AP radiograph showing Type 5 AVN developed after open reduction, VDRO, and pelvic osteotomy. (**c**) At 6 years after surgery, the femoral head was noted to be remodelled without subluxation.

**Figure 4:**
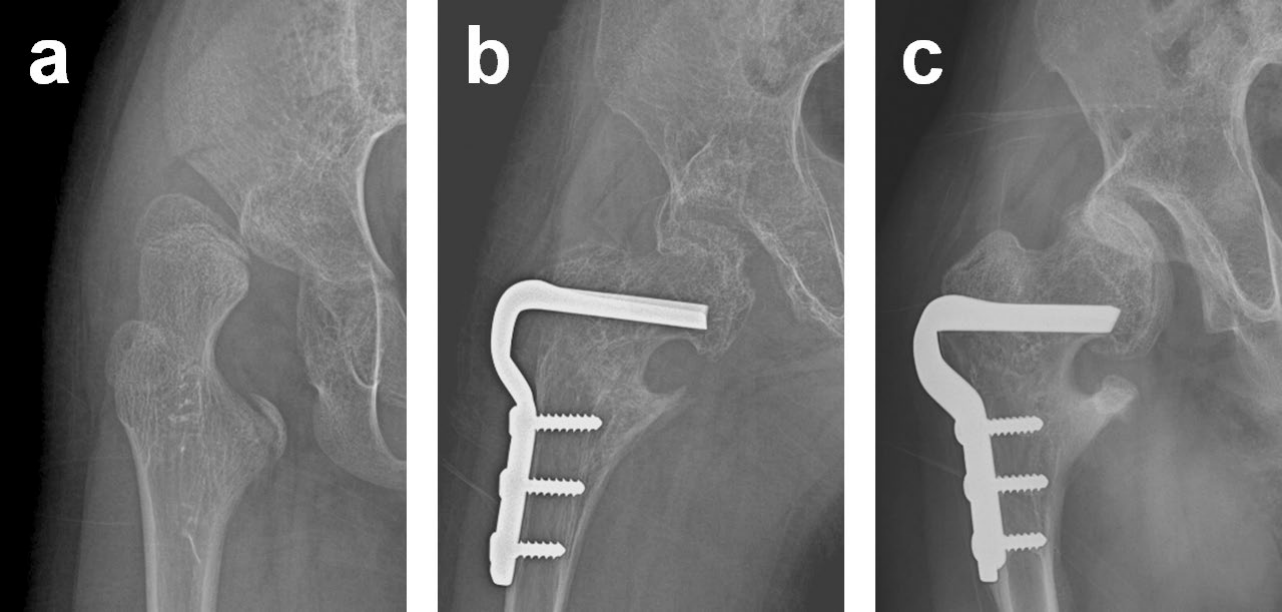
(**a**) Preoperative AP radiograph showing a dislocated hip in a 9-year-old girl. (**b**) AP radiograph showing Type 5 AVN developed after open reduction, VDRO, and pelvic osteotomy. (**c**) At 4 years after surgery, the femoral head failed to be remodelled and the hip was subluxated.

Remodelling of the femoral head was noted in 152 hips (89.9%) at the latest follow-up. The frequency of failed remodelling and unsatisfactory outcomes in relation to the type of AVN are summarized in Table 6. All hips with Types 1, 2 and 3 underwent successful remodelling and had similar rates of unsatisfactory outcomes as hips without any evidence of AVN (Fig. 2). Hips with Type 4 AVN had a higher incidence of unsatisfactory outcomes (22.2%) in comparison to those with Types 1, 2 and 3. This is despite the fact that all femoral heads with Type 4 AVN experienced successful remodelling, except two (96.8%). The highest incidence of unsatisfactory outcomes (34.5%) was seen in Type 5 hips, and the femoral heads were found to be remodelled in 48.3% of these cases (Fig. 3). There was no statistical difference in preoperative spherical index among the groups (p = 0.171). The spherical index was noted to increase significantly at the latest follow-up in all hips except those with Type 5 AVN. Of the 41 hips with unsatisfactory outcomes, 27 hips experienced AVN; 24 (88.9%) of which were Type 4 or 5, and 3 were Type 1. Eight (47.1%) of the 17 hips with failed remodelling experienced unsatisfactory outcomes (Fig. 4), and the risk of unsatisfactory outcomes was found to increase significantly with failed remodelling of the head (OR 6.22, 95% CI 2.14 to 18.05, p = 0.001).

**Table 6:** Frequency of failed remodelling and unsatisfactory outcome in relation to the type of AVN.

Type of AVN	Number of hips	Spherical index (mean ± SD)			Failed remodelling (%)	Unsatisfactory outcome (%)
		Preoperative	Final follow-up	p value		
None	225	37.7 ± 4.6	44.3 ± 5.0	< 0.001		14 (6.2)
1	64	37.8 ± 4.0	45.0 ± 4.5	< 0.001	0 (0)	3 (4.7)
2	9	37.3 ± 4.7	46.7 ± 6.5	0.001	0 (0)	0 (0)
3	4	40.0 ± 3.6	45.5 ± 5.2	0.026	0 (0)	0 (0)
4	63	38.2 ± 5.3	42.2 ± 5.1	< 0.001	2 (3.2)	14 (22.2)
5	29	40.3 ± 5.4	38.1 ± 8.5	0.253	15 (51.7)	10 (34.5)

*AVN* avascular necrosis of the femoral head.

## Discussion

AVN following reconstructive hip surgery for CP has unfortunately only been mentioned as an incidence in the literature, with reported rates varying greatly from 0 to 68.7% ^4–9,13,25–27^. However, many have failed to explain their methodology for diagnosing AVN ^8,25–27^. Furthermore, whilst those that have, simply adopted different AVN classification systems for DDH ^5–7,9^. There are several classification systems used to assess AVN following the treatment of DDH ^1,28,29^. Nevertheless, such systems cannot be easily translated across as the two conditions differ greatly in their pathophysiology for hip displacement and the subsequent changes in bone and soft tissue around the hip joint. In addition, in comparison to CP hips, femoral heads in DDH are mainly composed of immature chondro-osseous tissues at the time of reduction. Therefore, all the above-mentioned suggests that spastic hips complicated with AVN may result in differing hip characteristics and natural history.

Our study has several limitations. First, the retrospective nature of our study may result in selection and/or transfer bias. Some patients did not reach skeletal maturity at the latest follow-up and future re-migration of the femoral head may be possible due to the nature of the spastic disease. However, it should be noted that for all hips enrolled in the study we were able to determine their remodelling. Second, the ultimate goal of hip reconstructions in CP is to provide the patients with an improved quality of life. The migration percentage is a valid and reliable parameter to quantify hip displacement in patients with CP and is known to be associated with functional limitations ^19,30^. Furthermore, a migration percentage of more than 30% is related with increased pain ^31,32^. However, it would still be of great interest to have longer follow-up periods and to study functional aspects related to the hips complicated by AVN such as pain scores, sitting duration, and range of hip motion.

In this study we proposed a classification system to clearly recognize the types of AVN experienced in CP patients and investigated these types for their clinical relevance and association with the fate of these hips. In our study, femoral heads without loss of its epiphyseal height were classified as having Type 1 AVN; this may correspond to the lateral pillar classification group A in Perthes disease ^33^ and also represent the definition of AVN in other previous studies ^8,26,27^. We identified a high incidence of AVN and the majority involved the entire epiphysis. Additionally, we found that older age at the time of reduction, higher preoperative migration percentage, and open reduction procedures were significantly associated with the development of AVN. Severely displaced hips are more susceptible to vascular insults during surgical reduction and increased intra-articular pressure may ensue after relocation of a femoral head ^13^. Both the greater frequency of entire epiphyseal involvement and overall high incidence rates of AVN may be interpreted from the viewpoint that larger numbers of severely displaced hips necessitating open reduction were included in our cohort ^15,34^. Our findings are in line with Koch et al.’s ^6^ series reporting a higher incidence of 68.7% of AVN in their cohort with a mean preoperative migration percentage of more than 80%.

The question that may arise when discussing outcomes in spastic hips with AVN may be; whether the final head shape is influenced by the damage incurred prior to treatment from the forces involved in a subluxated or dislocated position or by the postoperative ischemic insult or a combination of both? AVN affecting the entire epiphysis with a collapsed height greater than 50% were significantly related with failed remodelling of the femoral head. Despite this result, we believe that a conclusion cannot be made that AVN is solely responsible for remodelling and we believe there is a complex interplay of multiple mechanisms that determine the final head shape. One of which is the growth disturbances resulting from the spastic forces that flex and adduct the hip in the presence of weak hip abductors during growth ^10,35^. These muscle imbalances induce changes in the proximal femoral geometry as well as progressively displace the femoral head out of the acetabulum, and result in variable degrees of femoral head deformities.

Another possible mechanism affecting the remodelling of AVN may be the effects from damaging the subcapital growth plate. Unfortunately, we were unable to detect a consistent pattern of radiologic changes that were indicative of physeal growth disturbances. We believe that it would not be possible to ascertain whether any consequences of physeal growth disturbances are from postoperative AVN occurred in the physis or due to persistent abnormal forces exerted in a chronically displaced hip. A minor rebound of the head-shaft angle was observed at the final follow-up, however, we do not know whether this is due to the retarded growth at the lateral aspect of the growth plate or by persistent weakness of the hip abductors that may result in the recurrence of coxa valga. Further follow-up of our patients will be necessary in order to understand all the above-mentioned complex interactions and the exact role of AVN in the final shape of the femoral head and remodelling.

Nevertheless, it is worthwhile monitoring the remodelling status of the femoral head after the development of AVN in CP. This is because the dysplastic acetabulum has limited capabilities in accommodating for alterations in the shape of femoral head. Recent studies have suggested that remodelling of the aspherical femoral head may occur after surgery in CP ^36,37^. However, these implications are limited in that they did not examine serial radiographs during follow-up. Despite the attempt of standardized techniques for imaging in quadriplegic CP patients, preoperative radiographs are limited in that they might not accurately delineate the real shape of the femoral head due to the often displaced and rotated nature of the spastic hip. Furthermore, very few have investigated the relationship between remodelling and AVN, making it difficult to determine its influence on outcomes. Exacerbating this, in a recent study investigating remodelling, the authors simply stated the incidence without outlining any clear methodology of recognition ^7^. Rutz et al. ^14^ reported that 6.6% of their hips demonstrated early radiographic progression of a femoral head deformity and subsequent delayed remodelling that took up to 2 years to complete. However, they did not give any further consideration to these findings, and we believe their observations were related to AVN following hip reconstructions.

In order to objectively analyze the incidence of hip remodelling with respect to each type of AVN, we systematically evaluated the femoral head shape with use of the spherical index and Mose criteria. In contrast to cases with total involvement of the epiphysis and severe height loss, hips that had changes confined to the lateral part of the epiphysis (Types 2 and 3) and those with mild to moderate changes to the entire epiphysis (Types 1 and 4) experienced successful remodelling of AVN. In addition, the spherical index was increased in hips with Types 1, 2, 3, and 4 AVN at the latest follow-up and this increase was comparable to the hips that did not experience any AVN. Therefore, our results also indicate that as long as the hip is not affected by a severe type of AVN, the overall sphericity of the femoral is improved after hip reconstructions in CP.

We interpret our findings to mean that unsatisfactory outcomes are more likely to arise when the femoral head complicated by AVN fails to restore its epiphyseal height to its preoperative state. This is especially the case in younger patients whose femoral heads were not deeply relocated within the acetabulum. This may be explained by the fact that hips treated with VDRO at an earlier age are more likely to have a recurrence of coxa valga and subsequent hip displacement ^38,39^. Furthermore, younger hips that are complicated by severe forms of AVN are more vulnerable to poor regeneration ^1,3^, especially when these hips have failed to achieve concentric reduction, which is a prerequisite for normal hip development. On the other hand, contrary to previous studies ^14,15^, the preoperative migration percentage was not found to be a factor affecting the radiologic outcomes and this may be accounted for by the inclusion of AVN in our multivariable analysis.

In conclusion, AVN was a common complication after hip reconstructions in nonambulatory patients with spastic CP. However, it was not necessarily associated with poor hip development and depending on the severity and location, was a prognostic factor for unsatisfactory outcomes. Additionally, we found that AVN was remodelled and overall femoral head sphericity improved in hips that have not experienced a severe loss of epiphyseal height. Future studies will be needed to correlate the radiological findings and the patients’ function and quality of life.

## Data Availability

The data that support the findings of this study are available from the corresponding author upon reasonable request.
